# Revealing the sensory impact of different levels and combinations of esters and volatile thiols in Chardonnay wines

**DOI:** 10.1016/j.heliyon.2023.e12862

**Published:** 2023-01-07

**Authors:** Angelica Iobbi, Yanming Di, Elizabeth Tomasino

**Affiliations:** a Department of Food Science and Technology, Oregon State University, Corvallis, OR, 97331, USA; b Department of Statistics, Oregon State University, Corvallis, OR, 97331, USA

**Keywords:** Esters, Volatile thiols, Sensory descriptive analysis, Check-all-that-apply, Tropical fruit aroma, Aroma interaction

## Abstract

The assessment of different aroma families on tropical fruit aroma perception is still not well understood. This study aimed to investigate the effect of esters and volatile thiols on tropical fruit aroma perception in white wines. Four levels of thiols (none, low, medium and high) and three levels of esters (none, low, medium) were added to a dearomatized white wine base in a full factorial design. Check-All-That-Apply (CATA) was used to determine the aroma descriptors that most differentiated the wines followed by Sensory Descriptive Analysis (SDA) to evaluate the intensity of those significant aroma attributes. More than 78% of the total variance was described in the first two dimensions when using Canonical Variate Analysis. Tropical fruit aromas were associated with wines containing different levels of esters and ester-thiol combinations. Volatile thiols alone imparted an earthy aroma and were grouped with the control wine. The different ester-thiol combinations altered the tropical fruit aroma quality in the wines from citrus to passionfruit, pineapple and guava. Understanding the cause of tropical fruit aroma allows for targeted processing to achieve the desired wine sensory quality.

## 1 Introduction

Consumers with wine knowledge tend to make their purchase decisions based on wine sensory qualities, such as aroma, taste and mouthfeel [1]. Tropical fruit aromas, such as passionfruit, guava and grapefruit are present in a wide array of white wines and studies revealed that these aromas are well accepted by consumers [2,3]. Thus, from a flavor chemistry standpoint, it is crucial to uncover the volatile compounds and possible aroma combinations that are associated with the perception of tropical fruit aroma in wine, particularly when trying to achieve this quality each year.

Traditionally, the compounds thought to cause tropical fruit aromas are volatile thiols 3-mercaptohexan-1-ol (3-MH), 3-mercaptohexyl acetate (3-MHA) and 4-mercapto-4-methylpentan-2-one (4-MMP) [[4], [5], [6]]. These sulfur compounds were identified in Sauvignon blanc wines, known for its strong tropical fruit aroma [7,8]. In Sauvignon blanc, the total concentrations of these compounds are very high, ranging from 25.8-7,256 ng/L for 3-MH, 6.7-591 ng/L for 3-MHA and <0.6-87.9 for 4-MMP [9].

Volatile thiols are found in trace concentrations in most wines (excluding Sauvignon blanc) and have extremely low perception thresholds (Table 1). There is substantial evidence suggesting that the volatile thiols are key contributors to tropical fruit and green aromas in wines [3,9,10]. But other work has shown the presence of these compounds in additional grape varieties, including Chardonnay, Gewürztraminer, Riesling, Muscat, Verdejo, at concentrations much lower than those measured in Sauvignon blanc [2,8,9].

**Table 1:** Levels, concentrations, and thresholds of esters and thiols spiked to the wines.

	Control	LE	ME	LT	MT	HT	CAS#	Threshold (μg/L)
Ethyl butanoate	**120**	944.17	1770.0				105-54-4	400^1^
Ethyl hexanoate	0.35	56.13	111.90				123-66-0	14^2^
Ethyl octanoate	0.90	52.98	104.07				106-32-1	580^1^
Ethyl decanoate	0.20	2.26	4.32				110-38-3	20^2^
Ethyl 2-hexenoate		4.95	29.71				1552-67-6	0.02^3^
Ethyl 2-methylpropanoate	**5**	**7.52**	**10.04**				97-62-1	15^4^
Ethyl 2-methylbutanoate	10	15.01	20.02				7452-79-1	1^5^
Ethyl 3-methylbutanoate	15	22.53	30.07				108-64-5	3^2^
Ethyl acetate	30	44	58				141-78-6	12000^1^
Hexyl acetate		**237.60**	**475.20**				142-92-7	670^1^
2-phenylethyl acetate	400	400	400				103-45-7	250^5^
2-methylpropyl acetate	**15**	**41.93**	**68.86**				110-19-0	1600^6^
3-methylbutyl acetate	2	3.39	4.78				123-92-2	160^1^
3-SH				**3.62**	119.22	241.33	51755-83-0	0.06^7^
3-SHA				**1.81**	5.37	10.88	136954-20-6	0.004^8^
4-MMP				5.88	17.19	34.8	19872-52-7	0.0008^9^

LE = low ester, ME = medium ester, LT = low thiol, MT = medium thiol, HT = high thiol.

Numbers in bold refer to compounds which concentrations are below threshold perception.

1 Peinado et al. 2004;

2 Ferreira et al. 2000;

3 Berger et al. 1985;

4 Juan et al. 2012;

5 Guth 1997;

6 Ferreira et al. 2002;

7 Tominaga et al. 1996a;

8 Tominaga et al. 1996b;

9 Darriet et al. 1995.

While much work links volatile thiols to tropical fruit aromas, it has been found that wines that have low or no volatile thiol compounds can also have tropical fruit aromas [10], suggesting that other compounds may be the cause of this aroma, with fermentation esters being a possibility [3]. Esters are compounds found in almost all wines, although the type of esters and concentrations vary [11]. It is thought that esters as a family of compounds may be responsible for the fruity aromas of wine [12] and ethyl esters have been linked to tropical fruit aromas in white wines [13]. The aroma compounds that are found in tropical fruits include a group of tropical fruits characterized by terpenes, a group characterized by sulfur compounds and a group characterized by esters [14]. Therefore, it is extremely likely that there are multiple aroma compounds that can cause tropical fruit aromas in wines, not just volatile thiols.

Therefore, considering the potential desirability of tropical fruit aroma to certain white wine styles, especially in wines that have low or total absence of thiol precursors in the grape, the objective of this work was to determine the cause of tropical fruit aroma in Chardonnay wines, focusing on volatile thiol and fermentation ester compounds. The hypothesis of this study is that both esters and volatile thiols in combination are the cause of tropical fruit aromas in white wines, at the concentrations found in Chardonnay wines. It is important to not only focus on the individual compounds but also different combinations of compounds as research has shown that when aroma compounds are in mixtures, the perceived aromas can be different than the aromas normally associated with the individual compounds [12,15]. While volatile thiols and esters have been associated with tropical fruit aroma in white wines [2,9,13], when they are combined, the resulting aroma may be something other than tropical fruit [16].

## 2 Material and methods

### 2.1 Chemicals

Acetaldehyde (75-07-0) (98%), 2,3-butanedione (431-03-8) (97%), hexan-1-ol (111-27-3) (98%), 3-(methylthio)-1-propyl alcohol (505-10-2) (98%), 2-methylpropan-1-ol (78-83-1) (99%), butyric acid (107-92-6) (99%), decanoic acid (334-48-5) (98%), 2-methylpropanoic acid (79-31-2) (99%), 2-methylbutanoic acid (116-53-0) (98%), 3-methylbutanoic acid (503-74-2) (99%), hexanoic acid (142-62-1) (99%), octanoic acid (124-07-2) (99%), 2-methylpropyl acetate (110-19-0) (99%), 2-phenylethyl acetate (103-45-7) (98%), ethyl 3-methylbutanoate (108-64-5) (98%), ethyl 2-methylpropanoate (7452-79-1) (99%), ethyl butanoate (105-54-4) (99%), ethyl decanoate (110-38-3) (99%), ethyl hexanoate (123-66-0) (99%), ethyl octanoate (106-32-1) (99%), 3-methylbutyl acetate (123-92-2) (99%), hexyl acetate (142-92-7) (99%), 3-mercaptohexan-1-ol (51755-83-0) (≤100%), 3-mercaptohexyl acetate (136954-20-6) (≥98%), 4-methyl-4-sulfanylpentan-2-one (19872-52-7) (≤100%) were obtained from Millipore Sigma (St. Louis, MO, USA). Ethyl 2-hexenoate (1552-67-6) (>97%) was purchased from Tokyo Chemical Industry Co., Ltd. (Portland, OR, USA). Acetic acid (64-19-7) (99.7%) was obtained from VWR International, LLC (Radnor, PA, USA). Ethyl acetate (141-78-6) (99.9%) was purchased from Fisher International Scientific, Inc. (Hampton, NH, USA). Milli-Q water was obtained from a Millipore Continental water system (EMD-Millipore, Billerica, MA, USA). HPLC-grade ethanol (64-17-5) (100%) was obtained from Pharmco-AAPER (Vancouver, WA, USA). LiChrolut® EN was obtained from Millipore Sigma (St. Louis, MO, USA).

### 2.2 Wine base

Pinot gris wine, vintage 2019, was made by standard winemaking procedures. Grapes were harvested at the OSU Woodhall Vineyard and were processed at OSU Research Winery. Grapes were destemmed, crushed and pressed at 1 bar for 18 min. The juice (°Brix: 23.5, pH: 3.17, titratable acidity: 5.26 g/L) was racked-off and Fermaid-K (0.25 g/L) (Lallemand, Inc., Montreal, Quebec, Canada) and *Saccharomyces cerevisiae* yeast Lalvin V1116 (0.25 g/L) (Lallemand, Inc., Montreal, Quebec, Canada) were added using standard rehydration protocols according to manufacturers instructors. The wine was fermented at 21 °C ± 2 to dryness in a 600 L stainless steel tank. After fermentation was complete, <0 °Brix, bentonite was added in excess at approximately 0.12 g/L to reduce the volatile composition of the wine [17]. The wine was topped up to 30 mg/L sulfur dioxide and filtered through a one μm nylon cartridge filtration (G.W. Kent, MI, USA) prior to bottling in 375 mL bottles with screw caps (Amcor, Zürich, Switzerland). The wine (ethanol: 11.5% (v/v)) was stored at 4 °C until used. The Pinot gris wine was dearomatized using Licholut® EN (2 g/L), stirred for 14 h and filtered through a Whatman 413 filter, seven days prior to the sensory panel. This amount was able to remove all the compounds in the wine to undetectable levels (data not shown, 18).

### 2.3 Panelists

A total of 49 white wine consumers (26 F, 22 M and one non-binary) participated in Check-All-That-Apply (CATA) and 13 trained panelists (6 F, 7 M) participated in Sensory Descriptive Analysis (SDA). Subjects age ranged between 21 and 60+ years old in both studies. General participation criteria required all subjects to consume at least one glass of white wine once a week, be over 21 years old, speak English, not be sick, smoke and have any diagnosed smell and taste disorders, not be pregnant and/or nursing (female subjects) and not have wine allergy. Approval for work was granted by the Institutional Review Board (#8606) at Oregon State University. Approved consent was obtained for all participants for all sensory studies.

### 2.4 Stimuli

Standards of 1 g/L of each compound were made in 100% ethanol and then combined in 14% (v/v) aqueous ethanol solutions to reach specific concentrations when added to the wine (Table 1). All standard solutions were stored at −20 °C until day of use. A combination of acetate and ethyl esters, higher alcohols and volatile acids, which are normally metabolized by yeast during white wine fermentation and found normally in all white wines, were added to the dearomatized wine to form the aroma base [16,19] (Table S1). The aroma base was added to the dearomatized wine approximately 12 h prior to sensory analysis and stored at 4 °C. The wine was poured into 375 mL bottles and treatment aromas were added to the bottles in the morning of sensory analysis (Table 1). The control wine (aroma base), three levels of esters (none, low and medium concentrations) and four levels of thiols (none, low, medium and high concentrations) were added to the treatment wines in a full factorial design, resulting in 12 samples for sensory analysis (Table 2). All compounds added to the dearomatized wine were added at concentrations found in Chardonnay wines. The wines were stored at 4 °C and brought to room temperature (21 °C ± 2) approximately 1 h before each sensory session. A new treatment bottle was open before each session. The treatment bottles were inverted three times before pouring to ensure that all volatiles were integrated with the wine matrix. The concentration of the compounds added to the treatment wines were from published literature [2,20].

**Table 2:** Ester and thiol levels added to the wines.

	Aroma base	LE	ME	LT	MT	HT
T1 (control)	x					
T2	x	x				
T3	x		x			
T4	x			x		
T5	x				x	
T6	x					x
T7	x	x		x		
T8	x	x			x	
T9	x	x				x
T10	x		x	x		
T11	x		x		x	
T12	x		x			x

### 2.5 Sensory analysis

20 mL of each wine treatment was poured approximately 30 min before testing (Table 2). Samples were served at room temperature (21 °C ± 2) in standard ISO tasting black glasses [35], covered with PET plastic cup lids (Choice Foodservice Products, China) and labeled with random three-digit codes. Panelists assessed the samples in individual booths under a mix of natural and artificial light. The ambient temperature was 21 °C ± 2 °C and air purifiers (Winix Inc., Vernon Hills, IL, USA) were used to eliminate possible odors. Panelists were asked to evaluate the orthonasal aroma of the samples in both CATA and (SDA) studies. Samples were presented in a monadic order. Sensory analysis took place at the Arbuthnot dairy lab at Oregon State University. CATA panels were carried out in June 2019 and the SDA panel was carried out in September 2020. All tests were performed using Qualtrics online software (Qualtrics^XM^, Provo, UT).

### 2.6 Check-all-that-apply

24 terms were given in the CATA analysis (Table S2). The terms were selected from literature to span the sensory space of fruity attributes related to ester and thiol compounds in white wines [2,3,21]. The order of which each CATA term appeared for each subject was randomized across samples [22]. To avoid sensory fatigue, consumers assessed the samples in two sessions: 25 subjects evaluated order #1 and 24 individuals evaluated order #2 (Table S3). Both sessions were carried out within the same week. A forced 1-min break in between samples was applied in all sessions.

### 2.7 Sensory descriptive analysis

The trained panel consisted of students and staff members from Oregon State University and Corvallis community members that regularly participate in SDA studies. First, panelists participated in three training sessions of 1 h each to become familiarized with the aroma and intensity of the selected attributes as described in Tomasino et al. [16]. In the first two training sessions, subjects were asked to smell 10 aroma standards (pome, citrus, stone fruit, pineapple, mango, grapefruit, passionfruit, guava, grass and earthy) and select from multiple choice questions, the correct aroma and picture associated with that term (Table S4). At the end of the first two training sessions, panelists also evaluated the intensity of the 10 aroma attributes in two commercial wine samples on 10 cm unstructured line scales with indented anchor points (none-extreme). The terms stone fruit and mango were excluded for the third training session as panelists were confusing these terms with pome and passionfruit, respectively, in the multiple-choice questions. In the third training session, panelists evaluated the eight fruit standards and three commercial white wine samples that were selected for having overall fruity, green/grassy and tropical fruit aroma nuances, respectively on 10 cm unstructured line scales with indented anchor points (none-extreme). Panelists were also allowed to include two “other” terms for each sample in case the pre-selected terms did not fully describe the samples and to reduce any halo-dumping effects [23]. A replicate of one of the wines was included in the last session to evaluate panel repeatability [24]. The order of the samples and attributes were randomized across participants.

**Fig. 1:**
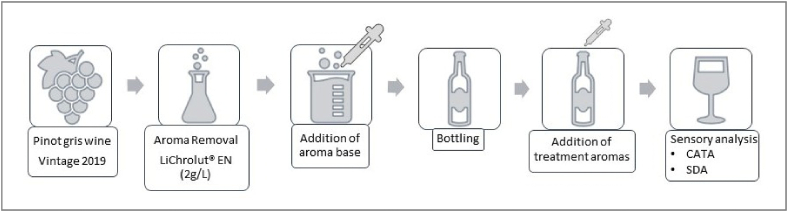
Workflow diagram of the wine aroma additions and sensory analysis procedures.

Testing samples were assessed in the week following training in duplicate over two sessions. In session one, seven panelists received order #1 and six panelists received order #2 (Table S3). In session two, the same seven panelists that had previously received order #1, received order #2 and the six other panelists received order #1. Both sessions were carried out within the same week. A forced 1-min break in between samples was applied in all sessions and another 5-min break was applied in between sets. As with the third training session, subjects were asked to mark on 10 cm unstructured line scales with indented anchor points (none-extreme) the intensity of eight aroma descriptors: citrus, earthy, grapefruit, grass, guava, passionfruit, pineapple and pome. The eight SDA descriptors were selected from the CATA results as they described the differences between samples. A workflow diagram showing the procedures used in this study is seen in Fig. 1.

### 2.8 Data analysis

For CATA data, a contingency table was built with the frequency of use of each attribute for each sample. Terms that were used less than 15% of their frequency were not retained for CA analysis [24]. Cochran’s Q test was used to evaluate the significant differences between samples based on the attributes. Post-hoc pairwise comparisons were made using the critical difference (Sheskin) procedure (p < 0.05) on the significant terms. Correspondence analysis (CA) was used to obtain the relationship between samples and attributes from CATA. SDA data was standardized for each descriptor by subtracting each data point by the mean and dividing by the standard deviation (χ − μ/σ). Linear mixed model was used to analyze the variance of the fixed effects for each aroma descriptor. The fixed effects of ester (three levels: none, low and medium) and thiol (four levels: none, low, medium and high) and their two-way interactions were evaluated. The effects of panelist and session were treated as random in this model. Sensory descriptors were considered significant if the associated p-value was <0.1. Panelist performance was evaluated by treating panelist and session as fixed effects. Canonical variate analysis (CVA) was conducted on the mean values of the significant (p < 0.1) attributes to determine which aroma descriptors were associated with the wine samples. For both CA and CVA data, hierarchical clustering (HC) followed by k-means clustering used to determine how different wines grouped [25]. All CATA and multivariate analyses for SDA were carried out using XLSTAT-Sensory annual version 2021.2.1.1129 (Addinsoft, Paris, France). Linear mixed models for SDA data were performed using RStudio version 2021.09.1 [39].

## 3 Results

### 3.1 Check-all-that-apply

**Fig. 2:**
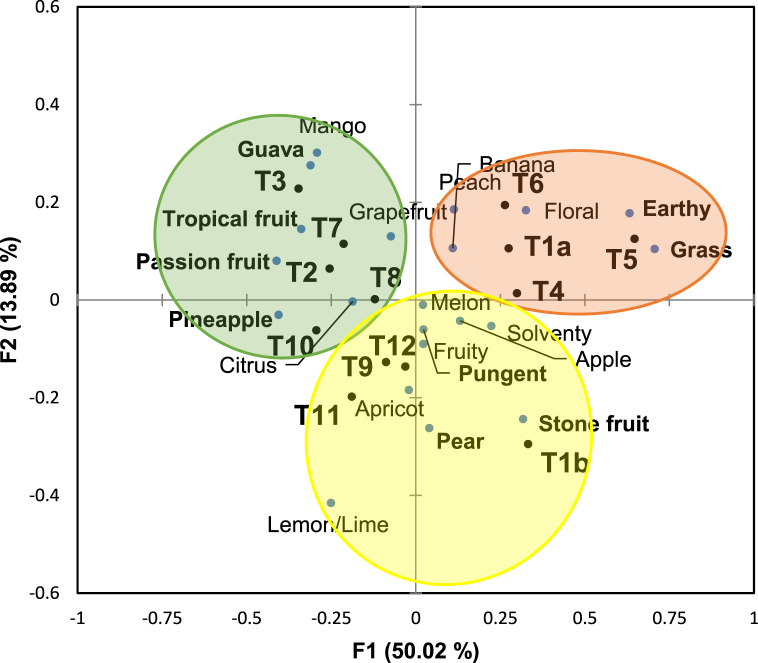
Separation of wines using correspondence analysis. Terms that were used less than 15% were removed. Cochran’s Q test significant terms (p < 0.05) are bolded in purple. Ellipses identify groups according to k-means clustering. (For interpretation of the references to colour in this figure legend, the reader is referred to the Web version of this article.)

Pineapple was the most frequently used term with 214 citations (Table S2). The terms orange, nectarine and vegetal were excluded from CA as their frequency of citation was less than 15% [24]. A total of 21 terms were retained for CA (Fig. 2). The terms pear, tropical fruit, pineapple, passionfruit, guava, stone fruit, vegetal, grass, earthy and pungent showed to be statistically different between treatment wines at various significance levels (Table 3). Looking at the superscripts from Sheskin procedure based on a 5% alpha level, wines T2, T3, T7 and T10 were significantly different from wines T1a and T5 for the term tropical fruit. For the pineapple descriptor, wines T2 and T3 and T7-T11 were statistically divergent from wines T4-T6. Moreover, T5 and T10 were statistically different for the term passionfruit. Finally, T5 was significantly different from wines T1b-T3, T7 and T9-T12 for the grass descriptor.

**Table 3:** Significance of main factors and their interactions for each sensory attribute by linear-mixed model.

Treatment		Pineapple	Citrus	Pome	Passionfruit	Guava	Grapefruit	Grass	Earthy	
T2	Low ester	***	ns	***	ns	ns	ns	ns	ns	
T3	Medium ester	**	**	***	*	ns	ns	ns	ns	
T4	Low thiol	ns	ns	ns	ns	ns	ns	ns	ns	
T5	Medium thiol	ns	ns	ns	ns	ns	ns	ns	**	
T6	High thiol	ns	ns	ns	ns	ns	ns	ns	**	
T7	Low ester:Low thiol	ns	*	ns	ns	ns	ns	ns	ns	
T8	Medium ester:Low thiol	ns	**	ns	ns	ns	ns	ns	ns	
T9	Low ester:Medium thiol	ns	ns	ns	ns	ns	*	ns	ns	
T10	Medium ester:Medium thiol	ns	ns	ns	ns	ns	ns	ns	ns	
T11	Low ester:High thiol	ns	ns	ns	ns	ns	ns	ns	ns	
T12	Medium ester:High thiol	ns	ns	ns	ns	*	ns	ns	**	

*p < 0.1; **p < 0.05; ***p < 0.01; ****p < 0.001.

The first two factors of the CA explained 63.91% of the variance (F1 = 50.02% and F2 = 13.89%, Fig. 2). The other factors did not add greatly to the total variance, so they were not considered for further statistical analysis. Along F1 axis, there was a clear separation of wines T1a and T1b and T4-T6 in the positive direction and wines T2 and T3 and T7-T12 in the negative direction. F2 separated wines T1a-T7 in the positive direction, from wines T1b and T9-T12 in the negative direction. Interestingly, wine T8 was centered in the vertical axis (Fig. 2).

K-means clustering showed that wines could be separated into 3 distinct groupings. Replicated controls were not in the same group but were the same across the F1 axis and differed by the F2 axis. T1a (control), T4-T6, the wine treatments with only thiols, were found in one group and associated with the terms earthy, grass, floral, banana and peach aromas. The wines with only esters (T2 and T3) were in a different group with T7, T8 and T10 and associated with the tropical fruit and citrus aromas. The last group contained T1b (control), T9, T11 and T12 and were associated with other fruity (pome fruits) and pungent aromas.

### 3.2 Sensory descriptive analysis

Panelist was significant (p < 0.05) for all the attributes, indicating that attribute scores differed among panelists when averaged over wine and session. Session, on the other hand, was not significant for any of the attributes (p < 0.05), which suggests that attribute scores between sessions did not differ when averaged over wine and panelist (data not shown). No significance was noted for each attribute for panelist*session*wine interaction, which shows panelists were consistent across each attribute, wine and session (data not shown) [26].

The main effects of low and medium esters were significant for pineapple and pome. Medium esters were also significant for citrus and passionfruit. In contrast, the medium and high levels of thiols were significant for the earthy descriptor. Significant interactions were found for citrus for low ester:low thiol and medium ester:low thiol, guava for medium ester:high thiol, grapefruit for low ester:medium thiol and earthy for medium ester:high thiol (Table 4).

**Table 4:** P-values for Cochran's Q test and posthoc differences for each aroma attribute.

Attributes	T1a	T1b	T2	T3	T4	T5	T6	T7	T8	T9	T10	T11	T12	P-values
Pear **	0.408 (b)	0.224 (ab)	0.204 (ab)	0.143 (a)	0.224 (ab)	0.163 (ab)	0.224 (ab)	0.245 (ab)	0.327 (ab)	0.367 (ab)	0.286 (ab)	0.367 (ab)	0.347 (ab)	0.010
Tropical fruit ***	0.082 (a)	0.184 (ab)	0.388 (b)	0.449 (b)	0.204 (ab)	0.061 (a)	0.224 (ab)	0.388 (b)	0.306 (ab)	0.306 (ab)	0.408 (b)	0.245 (ab)	0.245 (ab)	<0.0001
Pineapple ***	0.204 (abc)	0.204 (abc)	0.510 (d)	0.510 (d)	0.061 (a)	0.082 (ab)	0.122 (ab)	0.510 (d)	0.469 (cd)	0.469 (cd)	0.408 (cd)	0.469 (cd)	0.347 (bcd)	<0.0001
Passionfruit **	0.061 (ab)	0.082 (ab)	0.163 (ab)	0.163 (ab)	0.061 (ab)	0.020 (a)	0.041 (ab)	0.204 (ab)	0.184 (ab)	0.061 (ab)	0.224 (b)	0.102 (ab)	0.102 (ab)	0.004
Guava *	0.020 (a)	0.082 (ab)	0.143 (ab)	0.265 (b)	0.102 (ab)	0.041 (a)	0.122 (ab)	0.163 (ab)	0.122 (ab)	0.143 (ab)	0.143 (ab)	0.102 (ab)	0.082 (ab)	0.018
Stone fruit *	0.163 (b)	0.020 (a)	0.061 (ab)	0.041 (ab)	0.122 (ab)	0.082 (ab)	0.041 (ab)	0.082 (ab)	0.020 (a)	0.061 (ab)	0.061 (ab)	0.041 (ab)	0.061 (ab)	0.039
Vegetal **	0.102 (a)	0.061 (a)	0 (a)	0 (a)	0.041 (a)	0.082 (a)	0.082 (a)	0 (a)	0 (a)	0.041 (a)	0 (a)	0 (a)	0 (a)	0.002
Grass *	0.082 (ab)	0.020 (a)	0.020 (a)	0.041 (a)	0.082 (ab)	0.184 (b)	0.061 (ab)	0.020 (a)	0.082 (ab)	0.041 (a)	0.020 (a)	0.020 (a)	0.041 (a)	0.011
Earthy ***	0.245 (abc)	0.388 (c)	0.122 (ab)	0.061 (a)	0.224 (abc)	0.347 (bc)	0.327 (bc)	0.143 (ab)	0.143 (ab)	0.143 (ab)	0.020 (a)	0.061 (a)	0.163 (abc)	<0.0001
Pungent *	0.204 (ab)	0.367 (b)	0.224 (ab)	0.204 (ab)	0.265 (ab)	0.122 (a)	0.204 (ab)	0.143 (a)	0.184 (ab)	0.224 (ab)	0.327 (ab)	0.306 (ab)	0.245 (ab)	0.018

*, **, *** attributes are significant at p < 0.05, p < 0.01, and p < 0.001.

^NS^ attributes are not significant (p > 0.05).

Values with different superscripts within a row are significantly different from one another at p < 0.05 by the Critical difference (Sheskin) procedure.

**Fig. 3:**
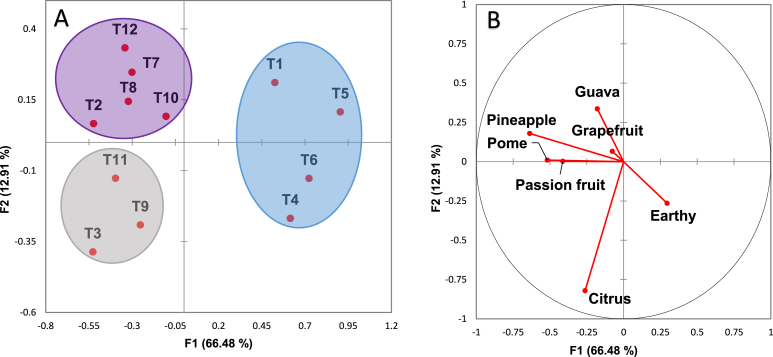
Separation of wines using discriminant analysis by F1 and F2. Profiles are positioned using the centroids for the wines. Scores are plotted in (A) and loadings for the sensory terms in (B). Ellipses identify groups according to k-means clustering.

The first two factors of the CVA plot explained 79.39% of the variance (F1 = 66.48% and F2 = 12.91%, Fig. 3). F1 separated wines T1 and T4-T6 in the positive direction and wines T2, T3 and T7-T12 in the negative direction. Wines T1, T2, T5, T7, T8, T10 and T12 were separated from wines T3, T4, T6, T9 and T11 by F2 (Fig. 3).

Three different groupings were identified with K-means clustering. Wine T1 and T4-T6 were grouped and associated with the earthy term. Wines T3, T9 and T11 were grouped and correlated with the citrus descriptor. A larger group of wines containing T2, T7, T8, T10 and T12 were associated with pome, pineapple, grapefruit, guava and passionfruit.

## 4 Discussion

Tropical fruit aroma is an important driver for consumer acceptance in Sauvignon blanc [3,10]. The volatile thiols 3-MH, 3-MHA and 4-MMP are well known for exclusively imparting these aromas in white wines [27,28]. However, recent studies have shown the perception of tropical fruit aroma in white wines which the presence of these varietal compounds are less prominent [16,20,29,30]. This brings to light the fact that other volatile compounds may also cause tropical fruit aroma. This study investigated if the combination of esters and volatile thiols, at concentrations found in Chardonnay wines, caused tropical fruit aroma.

While there is substantial evidence indicating that tropical fruit aroma is highly correlated to thiols and that such aroma is exclusively caused by these compounds [28], a similar outcome was clearly not observed in this study (Fig. 3). Our results showed that esters and ester-thiol combinations caused different qualities of tropical fruit aroma, including passionfruit, guava and grapefruit. This may be due to the concentrations evaluated in this study. Most studies investigate the sensory effect of thiols using concentrations found in Sauvignon blanc wines, which are generally much higher than the levels evaluated in this study. The concentrations in our study were taken from King et al. [3]. Levels of thiols found in white wines made from grape varieties other than Sauvignon blanc [31] have not been tested before and it is possible that they may result in different sensory perceptions.

Wines with only volatile thiols (T4-T6) caused earthy aromas in the wines (Fig. 2). This was an interesting result and presumably has not been yet demonstrated in wine. Earthy aroma has been normally linked to off-flavor compounds such as geosmin, 2-methylisoborneol, fenchol and fenchone [32] and the oxidation-related compound methional [33]. While earthy has not been used to describe thiol aromas in wine, “green” aroma was used to characterize 3-MH, 3-MHA and 4-MMP in several studies [2,3,9,21]. Moreover, although green was not significant in SDA, green and earthy were used to describe the same wines in the CATA procedure, which suggests that our results do agree with previous work.

Esters are widely known for contributing to the overall fruity profile of wines [12,34]. The results showed that wines with LE (T2) and LE:LT (T7) and LE:MT (T8) resulted in tropical fruit aromas. Medium esters in combination with LT (T10) and HT (T12) also resulted in tropical fruit aromas. All of these wines were found in the same cluster when using k-means clustering. However, by looking at the sensory vectors, T10 and T12 appear to be described as guava. T2 is most closely related to passionfruit sensory vector and T7 and T8 with pineapple and grapefruit. This suggests that low and medium esters are the cause of these tropical fruit aromas and the addition of the volatile thiols, alters the quality of the tropical fruit aroma. Previous work from Siebert et al. [21], has shown a relationship between some acetate and ethyl esters to tropical fruit aroma, when using concentrations found in Chardonnay and Viognier wines. Their result and our outcome suggest that in Chardonnay wines, tropical fruit aroma is caused by esters.

The other three wines (T3, T9 and T11) are found in a different cluster. This cluster does have some association with passion fruit as the entire cluster is in the negative F1 direction and the passionfruit vector characterizes wines in this direction. However, all three are also associated with the citrus vector, along the F2 axis. T3 contains medium esters and the other two are combinations of esters and thiols. The ester:thiol combinations resulting in a citrus aroma was interesting. Citrus has been correlated to different aroma families present in wine, such as esters [36], thiols [2,34,37] and terpenes [16,29,38]. Our results showing the cause was due to esters and thiols is in agreement with the previous work relating these compound families to this aroma.

Lastly, this study determined the cause of tropical fruit aromas in a very representative wine model, i.e. the use of the aroma base. The matrix that is used for reconstitution studies is very important for determining the aroma perceived. Tomasino et al. [16], showed that the usage of a simple wine matrix, wine matrix with nonvolatile compounds and wine matrix with volatile and nonvolatile compounds greatly alters perception of the aroma compounds of interest. The aroma base, or compounds found in all fermentations form the “vinous” aroma of wine [12]. In order to contribute to the aroma of wine then compounds must be able to break through this buffer [19]. Our usage of the aroma base in our studies provides the most realistic representation of aroma perception for wine.

## 5 Conclusion

Volatile thiols and fermentation esters impart distinct aromas in white wines. Volatile thiols alone did not contribute to tropical fruit aroma at the concentrations investigated in this work but instead provided earthy aromas. Low and medium ester concentrations, on the other hand, were associated with tropical fruit aroma. Two specific combinations of esters and thiols (LE:HT and ME:MT) resulted in citrus aromas. All ester-thiol combinations resulted in fruity aromas, which showed that thiols did not suppress the fruity aromas of esters. This work not only supports previous work investigating these compounds at these concentrations, but shows causation over correlation. To produce different styles of wines with or without these sensory qualities, winemakers can look to practices that alter the ester and thiol concentrations.

## References

[1] Corduas M., Cinquanta L., Ievoli C. (2013). The importance of wine attributes for purchase decisions: a study of Italian consumers' perception. Food Qual. Prefer..

[2] Capone D.L., Barker A., Williamson P.O., Francis I.L. (2018). The role of potent thiols in Chardonnay wine aroma: potent thiols in Chardonnay wine. Aust. J. Grape Wine Res..

[3] King E.S., Osidacz P., Curtin C., Bastian S.E.P., Francis I.L. (2011). Assessing desirable levels of sensory properties in Sauvignon Blanc wines - consumer preferences and contribution of key aroma compounds: consumer preference of Sauvignon Blanc flavours. Aust. J. Grape Wine Res..

[4] Buettner A., Schieberle P. (1999). Characterization of the most odor-active volatiles in fresh, hand-squeezed juice of grapefruit (*Citrus paradisi* Macfayden). J. Agric. Food Chem..

[5] Clery R.A., Hammond C.J. (2008). New sulfur components of pink guava fruit (*Psidium guajava* L.). J. Essent. Oil Res..

[6] Engel K.H., Tressl R. (1991). Identification of new sulfur-containing volatiles in yellow passionfruit (Passiflora edulis f. Flavicarpa). J. Agric. Food Chem..

[7] Darriet P., Tominaga T., Lavigne V., Boidron J.-N., Dubourdieu D. (1995). Identification of a powerful aromatic component of Vitis vinifera L. var. sauvignon wines: 4-mercapto-4-methylpentan-2-one. Flavour Fragrance J..

[8] Tominaga T., Furrer A., Henry R., Dubourdieu D. (1998). Identification of new volatile thiols in the aroma of Vitis vinifera L. var. Sauvignon blanc wines. Flavour Fragrance J..

[9] Mateo-Vivaracho L., Zapata J., Cacho J., Ferreira V. (2010). Analysis, occurrence, and potential sensory significance of five polyfunctional mercaptans in white wines. J. Agric. Food Chem..

[10] Lund C.M., Thompson M.K., Benkwitz F., Wohler M.W., Triggs C.M., Gardner R., Heymann H., Nicolau L. (2009). New Zealand sauvignon blanc distinct flavor characteristics: sensory, chemical, and consumer aspects. Am. J. Enol. Vitic..

[11] Etiévant (2017).

[12] Escudero A., Campo E., Fariña L., Cacho J., Ferreira V. (2007). Analytical characterization of the aroma of five premium red wines. Insights into the role of odor families and the concept of fruitiness of wines. J. Agric. Food Chem..

[13] Ferreira V., Fernández P., Peña C., Escudero A., Cacho J. (1995). Investigation on the role played by fermentation esters in the aroma of young Spanish wines by multivariate analysis. J. Sci. Food Agric..

[14] Lasekan O., Abbas K. (2012). Distinctive exotic flavor and aroma compounds of some tropical fruits and berries: a review. Crit. Rev. Food Sci. Nutr..

[15] Ferreira V., de-la-Fuente-Blanco A., Sáenz-Navajas M.-P. (2021). A new classification of perceptual interactions between odorants to interpret complex aroma systems. Application to model wine aroma. Foods.

[16] Tomasino E., Song M., Fuentes C. (2020). Odor perception interactions between free monoterpene isomers and wine composition of pinot gris wines. J. Agric. Food Chem..

[17] Vela E., Hernández-Orte P., Castro E., Ferreira V., Lopez R. (2017). Effect of bentonite fining on polyfunctional mercaptans and other volatile compounds in Sauvignon blanc wines. Am. J. Enol. Vitic..

[18] Tomasino E., Harrison R., Breitmeyer J., Sedcole R., Sherlock R., Frost A. (2015). Aroma composition of 2-year-old New Zealand Pinot Noir wine and its relationship to sensory characteristics using canonical correlation analysis and addition/omission tests. Aust. J. Grape Wine Res..

[19] Ferreira V., Sáenz-Navajas M., Campos E., Herrero P., del Fuente A., Fernández-Zubanp P. (2016). Sensory Interactions between six common aroma vectors explain four main red wine aroma nuances. Food Chem..

[20] Saberi S., Cliff M.A., Van Vuuren H.J.J. (2012). Impact of mixed S. cerevisiae strains on the production of volatiles and estimated sensory profiles of Chardonnay wines. Food Res. Int..

[21] Siebert T.E., Barker A., Pearson W., Barter S.R., de Barros Lopes M.A., Darriet P., Herderich M.J., Francis I.L. (2018). Volatile compounds related to ‘stone fruit’ aroma attributes in viognier and Chardonnay wines. J. Agric. Food Chem..

[22] Ares G., Jaeger S.R. (2013). Check-all-that-apply questions: influence of attribute order on sensory product characterization. Food Qual. Prefer..

[23] Clark C.C., Lawless H.T. (1994). Limiting response alternatives in time-intensity scaling: an examination of the halo-dumping effect. Chem. Senses.

[24] Campo E., Do B.V., Ferreira V., Valentin D. (2008). Aroma properties of young Spanish monovarietal white wines: a study using sorting task, list of terms and frequency of citation. Aust. J. Grape Wine Res..

[25] Pelonnier-Magimel E., Mangiorou P., Philippe D., De Revel G., Jourdes M., Marchal A., Marchand S., Pons A., Riquier L., Teissedre P.L., Thibon C. (2020). Sensory characterisation of Bordeaux red wines produced without added sulfites. OENO One.

[26] Tomasino E., Harrison R., Sedcole R., Frost A. (2013). Regional differentiation of New Zealand pinot noir wine by wine professionals using canonical variate analysis. Am. J. Enol. Vitic..

[27] Bonnaffoux H., Roland A., Schneider R., Cavelier F. (2021). Spotlight on release mechanisms of volatile thiols in beverages. Food Chem..

[28] Coetzee C., du Toit W.J. (2012). A comprehensive review on Sauvignon blanc aroma with a focus on certain positive volatile thiols. Food Res. Int..

[29] Chigo-Hernandez M.M., DuBois A., Tomasino E. (2022). Aroma perception of rose oxide, linalool and α-terpineol combinations in Gewürztraminer wine. Fermentation.

[30] Iobbi A., Tomasino E. (2021). Adapting polarized projective mapping to investigate fruitiness aroma perception of white wines from Oregon. Beverages.

[31] Tominaga T., Baltenweck-Guyot R., Peyrot des Gachons C., Dubourdieu D. (2000). Contribution of volatile thiols to the aromas of white wines made from several Vitis Vinifera grape varieties. Am. J. Enol. Vitic..

[32] Callejón R.M., Ubeda C., Ríos-Reina R., Morales M.L., Troncoso A.M. (2016). Recent developments in the analysis of musty odour compounds in water and wine: a review. J. Chromatogr. A.

[33] Coetzee C., Brand J., Emerton G., Jacobson D., Silva Ferreira A.C., du Toit W.J. (2015). Sensory interaction between 3-mercaptohexan-1-ol, 3-isobutyl-2-methoxypyrazine and oxidation-related compounds: sauvignon Blanc sensory interaction studies. Aust. J. Grape Wine Res..

[34] Ferreira V., Ortín N., Escudero A., López R., Cacho J. (2002). Chemical characterization of the aroma of Grenache rosé wines: aroma extract dilution analysis, quantitative determination, and sensory reconstitution studies. J. Agric. Food Chem..

[35] International Organization for Standardization (1977).

[36] Wang J., Capone D.L., Wilkinson K.L., Jeffery D.W. (2016). Chemical and sensory profiles of rosé wines from Australia. Food Chem..

[37] Tominaga T., Niclass Y., Frérot E., Dubourdieu D. (2006). Stereoisomeric distribution of 3-Mercaptohexan-1-ol and 3-mercaptohexyl acetate in dry and sweet white wines made from *Vitis vinifera* (var. Sauvignon blanc and semillon). J. Agric. Food Chem..

[38] Petronilho S., Lopez R., Ferreira V., Coimbra M.A., Rocha S.M. (2020). Revealing the usefulness of aroma networks to explain wine aroma properties: a case study of Portuguese wines. Molecules.

[39] R Core Team (2021). https://www.R-project.org/.

