# Supplementary material for: Revealing the sensory impact of different levels and combinations of esters and volatile thiols in Chardonnay wines
Source: Heliyon. 2023 Jan 7;9(1):e12862. doi: 10.1016/j.heliyon.2023.e12862 (PMC9860267; doi:10.1016/j.heliyon.2023.e12862)
Supplement: Multimedia component 1 [file mmc1.docx]

| **Table S1.** Concentration of the volatiles added to the aroma base | | |  |
| --- | --- | --- | --- |
| **Compounds** | **Concentration (µg/L)** | **CAS#** | **Threshold** |
| Acetaldehyde | 5000 | 75-07-0 | 500 |
| Butane-2,3-dione | 50 | 431-03-8 | 100 |
| Hexan-1-ol | 70 | 111-27-3 | 110 |
| 3- (methylthio)-1-propanol | 31 | 505-10-2 | 500 |
| 2-methylpropan-1-ol | 35000 | 78-83-1 | 4000 |
| Acetic acid | 100000 | 64-19-7 | 200000 |
| Butanoic acid | 650 | 107-92-6 | 10000 |
| Hexanoic acid | 1000 | 142-62-1 | 420 |
| Octanoic acid | 4000 | 124-07-2 | 500 |
| Decanoic acid | 200 | 334-48-5 | 1000 |
| 2-methylpropanoic acid | 800 | 79-31-2 | 200000 |
| 2-methylbutanoic acid | 100 | 116-53-0 | 3000 |
| 3-methylbutanoic acid | 700 | 503-74-2 | 3000 |
| Ethyl butanoate | 120 | 105-54-4 | 400 |
| Ethyl hexanoate | 350 | 123-66-0 | 0.014 |
| Ethyl octanoate | 900 | 106-32-1 | 0.58 |
| Ethyl decanoate | 200 | 110-38-3 | 0.2 |
| Ethyl 2-methylpropanoate | 5 | 97-62-1 | 15 |
| Ethyl 2-methylbutanoate | 10 | 7452-79-1 | 1 |
| Ethyl-3-methylbutanoate | 15 | 108-64-5 | 3 |
| Ethyl acetate | 30000 | 141-78-6 | 12000 |
| 2-phenylethyl acetate | 400 | 103-45-7 | 250 |
| 2-methylpropyl acetate | 15 | 110-19-0 | 1600 |
| 3-methylbutyl acetate | 2000 | 123-92-2 | 160 |
