# Supplementary material for: Revealing the sensory impact of different levels and combinations of esters and volatile thiols in Chardonnay wines
Source: Heliyon. 2023 Jan 7;9(1):e12862. doi: 10.1016/j.heliyon.2023.e12862 (PMC9860267; doi:10.1016/j.heliyon.2023.e12862)
Supplement: Multimedia component 2 [file mmc2.docx]

|  | | | | |  |  |  |  |  |  |  |  |  |  |
| --- | --- | --- | --- | --- | --- | --- | --- | --- | --- | --- | --- | --- | --- | --- |
| **Attributes** | **T1a** | **T1b** | **T2** | **T3** | **T4** | **T5** | **T6** | **T7** | **T8** | **T9** | **T10** | **T11** | **T12** | **Total** |
| Pineapple | 10 | 10 | 25 | 25 | 3 | 4 | 6 | 25 | 23 | 23 | 20 | 23 | 17 | 214 |
| Solventy | 17 | 17 | 8 | 9 | 17 | 18 | 13 | 14 | 16 | 17 | 14 | 13 | 14 | 187 |
| Pear | 20 | 11 | 10 | 7 | 11 | 8 | 11 | 12 | 16 | 18 | 14 | 18 | 17 | 173 |
| Tropical fruit | 4 | 9 | 19 | 22 | 10 | 3 | 11 | 19 | 15 | 15 | 20 | 12 | 12 | 171 |
| Fruity | 13 | 10 | 10 | 12 | 12 | 12 | 10 | 11 | 14 | 13 | 16 | 16 | 14 | 163 |
| Apple | 17 | 13 | 14 | 8 | 11 | 10 | 11 | 14 | 13 | 12 | 7 | 11 | 8 | 149 |
| Pungent | 10 | 18 | 11 | 10 | 13 | 6 | 10 | 7 | 9 | 11 | 16 | 15 | 12 | 148 |
| Honeydew | 7 | 7 | 9 | 7 | 13 | 8 | 12 | 13 | 13 | 12 | 11 | 10 | 14 | 136 |
| Earthy | 12 | 19 | 6 | 3 | 11 | 17 | 16 | 7 | 7 | 7 | 1 | 3 | 8 | 117 |
| Banana | 9 | 11 | 10 | 10 | 13 | 5 | 9 | 7 | 7 | 8 | 7 | 5 | 5 | 106 |
| Floral | 5 | 7 | 4 | 7 | 6 | 12 | 9 | 5 | 3 | 3 | 6 | 5 | 8 | 80 |
| Guava | 1 | 4 | 7 | 13 | 5 | 2 | 6 | 8 | 6 | 7 | 7 | 5 | 4 | 75 |
| Passion fruit | 3 | 4 | 8 | 8 | 3 | 1 | 2 | 10 | 9 | 3 | 11 | 5 | 5 | 72 |
| Citrus | 5 | 4 | 5 | 7 | 1 | 3 | 5 | 7 | 8 | 4 | 8 | 5 | 8 | 70 |
| Peach | 3 | 3 | 5 | 3 | 5 | 3 | 6 | 6 | 4 | 3 | 2 | 1 | 5 | 49 |
| Stone fruit | 8 | 1 | 3 | 2 | 6 | 4 | 2 | 4 | 1 | 3 | 3 | 2 | 3 | 42 |
| Apricot | 4 | 3 | 4 | 3 | 2 | 3 | 1 | 4 | 1 | 4 | 3 | 4 | 6 | 42 |
| Lemon/lime | 3 | 0 | 4 | 2 | 3 | 0 | 2 | 1 | 1 | 4 | 5 | 5 | 6 | 36 |
| Mango | 0 | 2 | 4 | 4 | 2 | 1 | 4 | 6 | 2 | 1 | 4 | 3 | 3 | 36 |
| Grass | 4 | 1 | 1 | 2 | 4 | 9 | 3 | 1 | 4 | 2 | 1 | 1 | 2 | 35 |
| Grapefruit | 0 | 1 | 3 | 4 | 2 | 4 | 2 | 3 | 2 | 4 | 1 | 5 | 2 | 33 |
| Orange | 0 | 0 | 0 | 3 | 1 | 2 | 2 | 2 | 2 | 4 | 3 | 2 | 1 | 22 |
| Vegetal | 5 | 3 | 0 | 0 | 2 | 4 | 4 | 0 | 0 | 2 | 0 | 0 | 0 | 20 |
| Nectarine | 1 | 1 | 1 | 3 | 3 | 0 | 1 | 2 | 1 | 0 | 0 | 2 | 3 | 18 |

**Table S2.** Contingency table built from CATA data
