# Supplementary material for: Revealing the sensory impact of different levels and combinations of esters and volatile thiols in Chardonnay wines
Source: Heliyon. 2023 Jan 7;9(1):e12862. doi: 10.1016/j.heliyon.2023.e12862 (PMC9860267; doi:10.1016/j.heliyon.2023.e12862)
Supplement: Multimedia component 3 [file mmc3.docx]

|  | | | | |
| --- | --- | --- | --- | --- |
|  | **Order #1** | | **Order #2** | |
|  | **Session/Set 1** | **Session/Set 2** | **Session/Set 1** | **Session/Set 2** |
| **T1 (control)** | x |  | x |  |
| **T2** | x |  | x |  |
| **T3** | x |  |  | x |
| **T4** |  | x | x |  |
| **T5** |  | x |  | x |
| **T6** |  | x |  | x |
| **T7** | x |  | x |  |
| **T8** | x |  | x |  |
| **T9** | x |  | x |  |
| **T10** |  | x |  | x |
| **T11** |  | x |  | x |
| **T12** |  | x |  | x |

**Table 3.** Sensory design showing the order that the samples were given to panelists in CATA and SDA panels
