# Supplementary material for: Revealing the sensory impact of different levels and combinations of esters and volatile thiols in Chardonnay wines
Source: Heliyon. 2023 Jan 7;9(1):e12862. doi: 10.1016/j.heliyon.2023.e12862 (PMC9860267; doi:10.1016/j.heliyon.2023.e12862)
Supplement: Multimedia component 4 [file mmc4.docx]

| **Table S4.** Standards used to train the Sensory Descriptive Analysis panel | | |  |
| --- | --- | --- | --- |
| **Attribute** | **Amount/glass*** | **Composition** | **Image** |
| Citrus | 1 tbsp. | 1:1 Lemom/lime whole fruit pureés | 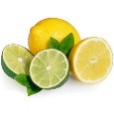 |
| Earthy | 1 tbsp. | All purpose garden soil (Miracle-Gro®) | 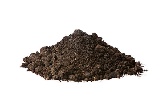 |
| Grapefruit | 1 tbsp. | Whole grapefruit pureé | 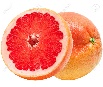 |
| Grass | 10 g | Fresh cut and chopped grass | 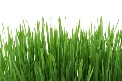 |
| Guava | 1 tbsp. | Guava pureé (The Perfect Purée®) | 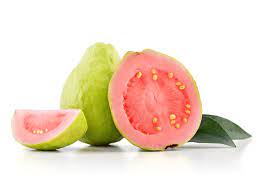 |
| Passionfruit | 1 tbsp. | Passionfruit pureé (The Perfect Purée®) | 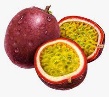 |
| Pineapple | 1 tbsp. | Peeled pineapple fruit juice | 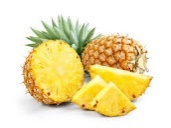 |
| Pome | 1 tbsp. | 1:1 Green apple and pear pureé (The Perfect Purée®) | 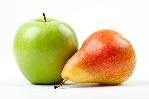 |
| *1tbsp = Approximately 15 g | |  |  |
