# Supplementary material for: Revealing the sensory impact of different levels and combinations of esters and volatile thiols in Chardonnay wines
Source: Heliyon. 2023 Jan 7;9(1):e12862. doi: 10.1016/j.heliyon.2023.e12862 (PMC9860267; doi:10.1016/j.heliyon.2023.e12862)

Example of CATA question:

There are a total of 2 sensory sessions for this study. Today is the first session.

From left to right, you will work your way through each sample, one at a time. Please evaluate **ONLY** one sample at a time and choose the descriptor(s) that best characterize(s) the sample. **Do not move on to the next sample until instructed to do so**.

More detailed instructions will be given later in the survey.

Please, **DO NOT TASTE** any of the samples.

Please, **NO CELLPHONE USE OR ANY OTHER FORM OF ENTERTAINMENT** for the duration of today's session.

If you have any questions, raise your hand and a server will assist you.

Instructions:
Please **smell** sample 534 and indicate which aromas you feel best describes the wine. There are no right or wrong choices, but try to answer to the best of your knowledge. You are allowed to check a maximum of 5 descriptors and a minimum of 1 descriptor.

☐ Apple	☐ Pineapple	☐ Stone fruit	☐ Citrus	☐ Grass
☐ Pear	☐ Passion fruit	☐ Apricot	☐ Lemon/Lime	☐ Earthy
☐ Melon/Honeydew	☐ Mango	☐ Peach	☐ Orange	☐ Pungent
☐ Floral	☐ Guava	☐ Nectarine	☐ Grapefruit	☐ Solventy
☐ Tropical fruit	☐ Banana	☐ Fruity	☐ Vegetal	☐ Other ________

Example of training question:

You will be partaking in 2 training sessions this week. In this first session, you will be presented with 10 different standards and 2 wines. From the given choices please choose the fruit that best describes the smell. You will then be provided with the actual fruit used and asked to re-smell the sample thinking about the fruit in an attempt to "train" you on that specific smell.

You are presented with three rows of samples. The first two rows are aroma standards and the last row contains the wines.

Directions for the wine samples will be given later in the survey.

Please do not taste any of the samples.

Please follow all directions given in the survey. If you have any questions please contact a server. You may now remove your face mask and start the sensory test.

Aroma standards questions:

Please remove the cover and smell sample #152. Choose the descriptor that best characterizes the smell.

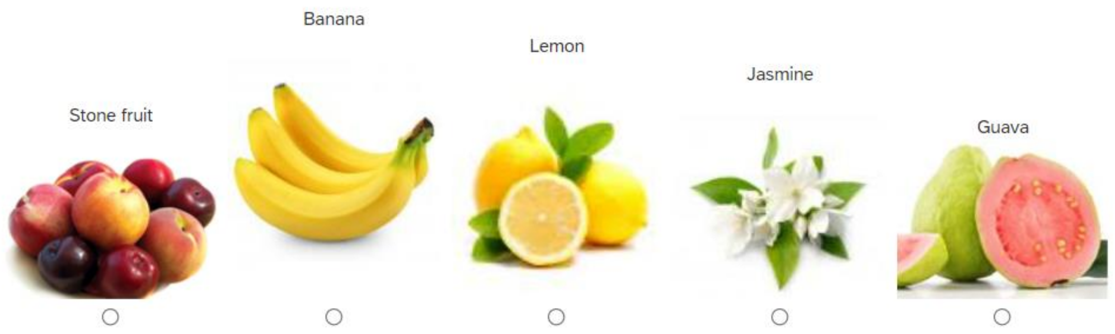

<u>Example of descriptive analysis question:</u>

Instructions:

What is the sample #?

Evaluate the aroma intensity for each descriptor. If it is not present then do not touch the slider or move it all the way to the left.

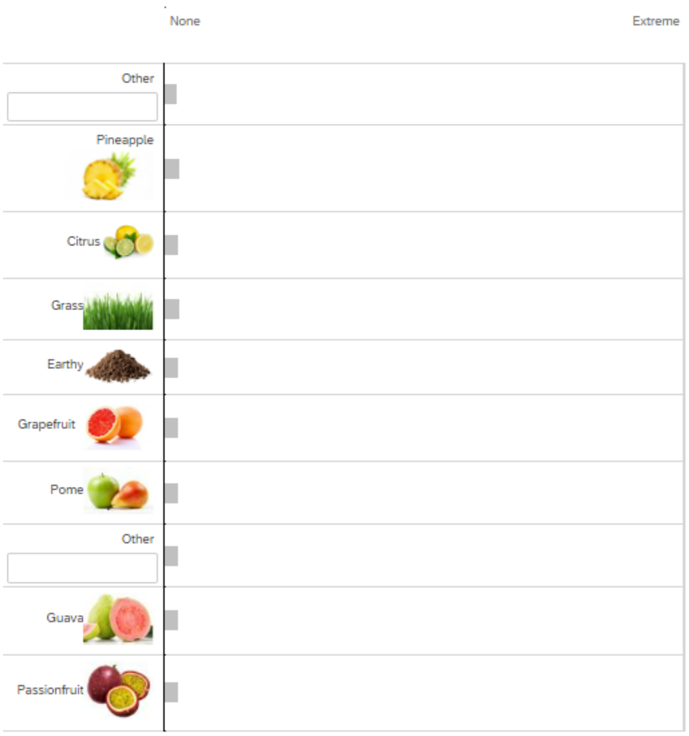

Supplement: Multimedia component 5 [file mmc5.pdf]
